# The Relationship between Dietary Fatty Acids and Inflammatory Genes on the Obese Phenotype and Serum Lipids

**DOI:** 10.3390/nu5051672

**Published:** 2013-05-21

**Authors:** Yael T. Joffe, Malcolm Collins, Julia H. Goedecke

**Affiliations:** 1 UCT/MRC Research Unit for Exercise Science and Sports Medicine, Department of Human Biology, University of Cape Town, 3rd Floor, SSISA, Boundary Rd, Newlands, Cape Town 7700, South Africa; E-Mails: yael.joffe@gmail.com (Y.T.J.); malcolm.collins@uct.ac.za (M.C.); 2 South African Medical Research Council, Francie van Zijl Drive, Parow vallei, Cape Town 7505, South Africa

**Keywords:** adipose tissue, dyslipidemia, SNP, dietary fat, inflammation, ethnicity

## Abstract

Obesity, a chronic low-grade inflammatory condition is associated with the development of many comorbidities including dyslipidemia. This review examines interactions between single nucleotide polymorphisms (SNP) in the inflammatory genes tumor necrosis alpha (*TNFA*) and interleukin-6 (*IL-6*) and dietary fatty acids, and their relationship with obesity and serum lipid levels. In summary, dietary fatty acids, in particular saturated fatty acids and the omega-3 and omega-6 polyunsaturated fatty acids, impact the expression of the cytokine genes *TNFA* and *IL-6*, and alter TNFα and IL-6 production. In addition, sequence variants in these genes have also been shown to alter their gene expression and plasma levels, and are associated with obesity, measures of adiposity and serum lipid concentrations. When interactions between dietary fatty acids and *TNFA* and *IL-6* SNPs on obesity and serum lipid were analyzed, both the quantity and quality of dietary fatty acids modulated the relationship between *TNFA* and *IL-6* SNPs on obesity and serum lipid profiles, thereby impacting the association between phenotype and genotype. Researching these diet–gene interactions more extensively, and understanding the role of ethnicity as a confounder in these relationships, may contribute to a better understanding of the inter-individual variability in the obese phenotype.

## 1. Introduction

Obesity, a chronic low-grade inflammatory condition, is associated with the development of many comorbidities, including cardiovascular disease (CVD), type 2 diabetes, and a number of cancers [1]. As more is understood about obesity, the complexity of this chronic disorder becomes more apparent, exhibiting a multi-factorial etiology [2]. Lifestyle factors such as diet and exercise continue to be recognized to play an important role in the development and progression of obesity and its comorbidities. However, genetic variation is also known to contribute to the obese phenotype. Lifestyle factors, including dietary components, such as fatty acids, interact with genetic variants to regulate the development and progression of obesity and its comorbidities. These complex interactions may explain differences observed in the obese phenotype and its comorbidities that vary both within and across populations [3,4,5,6].

**Figure 1:**
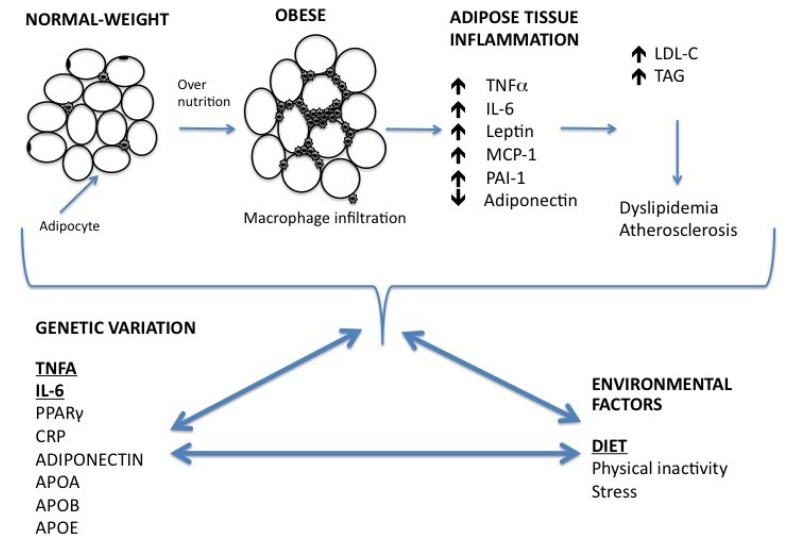
A proposed schematic diagram for obesity-associated low-grade inflammation, and the relationship of diet–gene interactions on obesity and dyslipidemia. Adipocytes become hypertrophic through over-nutrition. Expansion of adipose tissue in obesity leads to a subsequent increase in the production of chemokines by the adipocytes, resulting in increasing macrophage infiltration and enhanced production of pro-inflammatory cytokines, such as TNFα and IL-6. Obesity-associated low-grade inflammation results in an increase in serum trigycerides, and LDL-C concentrations and is associated with dyslipidemia. Environmental factors and DNA sequence variations in inflammatory genes, interact to impact molecular processes of the inflammatory pathway, serum lipids and the obese phenotype. *APOA*, Apolipoprotein A; *APOB*, Apolipoprotein B; *APOE*, Apolipoprotein E; *CRP*, C-reactive protein; IL-6, interleukin-6; LDL-C, low-density lipoprotein cholesterol; MCP-1, monocyte chemotactic protein-1; PAI, plasminogen activated inhibitor; *PPARγ*, peroxisome proliferator-activated receptor gamma; TAG, triglycerides; TNFα, tumor necrosis factor.

The aim of this review is to illustrate the current state of knowledge regarding the interaction between dietary fatty acids and cytokines associated with obesity and serum lipids. Dietary fatty acids modulate the regulation and production of tumor necrosis factor alpha (TNFα) and interleukin-6 (IL-6), thereby influencing inflammatory status. Furthermore, emerging research suggests that diet–gene interactions play an important role in the regulation of these inflammatory cytokines, impacting the obese phenotype. This review focuses on interactions between single nucleotide polymorphisms (SNPs) in the inflammatory genes *TNFA* and *IL-6*, and dietary fatty acids, and their relationship with obesity and serum lipid levels as proof-of-principle examples. Figure 1 illustrates the development of obesity-associated low-grade inflammation and the impact of diet–gene interactions on obesity and dyslipidemia.

## 2. Relationship between Inflammation, Obesity and Lipid Metabolism

White adipose tissue constitutes the majority of adipose tissue (AT) [7] and can be divided into two fractions; the adipocyte fraction composed of mature adipocytes, and the stroma vascular fraction (SVF) composed of many other cell types, including preadipocytes, macrophages, endothelial cells and fibroblasts [8]. Although AT is considered a metabolically active endocrine organ, the macrophages present in the SVF are the primary source of obesity-induced inflammation [9,10,11]. The number of macrophages present in the SVF is directly correlated with the level of adiposity and adipocyte size [10,12]. Adipocyte hypertrophy, results in increased chemokine secretion e.g., transforming growth factor β1, soluble ICAM, and monocyte chemotactic protein-1 (MCP-1) [10]. There is a subsequent increase in the infiltration of macrophages, which in turn secrete cytokines such as IL-6 and TNFα. Since AT expansion is characterized by increased macrophage infiltration, these cells are responsible for almost all TNFα and significant amounts of IL-6 secreted by AT [10].

Low-grade chronic inflammation also mediates all stages of atherosclerosis from initiation through to the complications of thrombosis. So much so, that elevated inflammatory markers are used to define risk of atherosclerotic complications, independent of myocardial damage [13]. One of the proposed mechanisms, linking inflammatory processes to the development of atheroma, is the low-density lipoprotein (LDL) oxidation hypothesis [14]. LDL in the intima, the innermost layer of an artery or vein, undergoes oxidative modification, inducing expression of proinflammatory cytokines, chemokines and other mediators of inflammation. Very-low density lipoprotein (VLDL) and intermediate-density molecules are also believed to undergo oxidative modification and may themselves activate the inflammatory processes of the vascular endothelial cells [15] through activation of nuclear factor kappa-light-chain-enhancer of activated B cells (NFκB) [16].

Genetic sequence variants—known as polymorphisms—within the promoter region of inflammatory genes influence gene transcription, altering protein production. Functional polymorphisms have been reported in the *TNFA*, *IL-1*, *IL-6* and *lymphotoxin-*α (*LTA*) inflammatory genes, altering cytokine production [17,18,19,20]. These polymorphisms have been shown to interact with dietary fatty acids to regulate production and secretion of cytokines, predisposing an individual to inflammation and altering obesity and associated comorbidity risk [6,21].

In a recent review article by Phillips [22], it has been suggested that inconsistencies in previous studies on the influence of cytokine polymorphisms in the risk of obesity, diabetes and the metabolic syndrome (MetS), may be in part explained by the fact that cytokines act in a complex network, and that studying single genes may not provide full insight [22]. The recent LIPGENE-SU.VI.MAX MetS case control study reported by Phillips *et al.*, examined the relationship between *LTA*, *IL-6* and *TNFA* gene variants and MetS [23]. They reported that the G allele of the *TNFA* –308 G > A SNP and the minor A allele of the *LTA* rs915654 SNP were associated with a 20%–40% higher MetS risk [23], but the combined effect of carrying both risk genotypes further increased MetS risk. It was also shown that total plasma PUFA/SFA levels modified the observed additive genetic effects of *IL-6*, *TNFA* and *LTA* [23], highlighting the importance of studying nutrigenetic SNP-SNP or gene-gene interactions.

## 3. Dietary Fatty Acids and Inflammation

Dietary fatty acids have received considerable attention for their ability to regulate inflammatory gene expression and secretion. It has been proposed that dietary fatty acids affect inflammatory processes through the modulation of transcription factors such as NFκB and peroxisome proliferator-activated receptor gamma (PPARγ) [24,25]. PPARγ inhibits NFκB, and both transcription factors are sensitive to dietary fatty acids [26].

There is general agreement that increasing dietary SFA intake, especially in overweight or obese individuals, is associated with raised inflammatory markers [27], predominately by activating the toll-like receptor 4 (TLR4) pathway. The TLR4 pathway is expressed in both subcutaneous adipose tissue (SAT) and visceral adipose tissue (VAT). SFAs serve as ligands for TLR4, inducing inflammatory changes in both adipocytes and macrophages through NFκB activation [28], increasing adipocytokine gene expression and production [29,30]. It has been shown that when adipocytes are exposed to the SFA, palmitic acid, IL-6 mRNA expression and protein production increased, most likely through activation of NFκB [31,32]. Similarly, monocytes were directly activated when exposed to SFA, especially lauric acid [30,33].

The *n*-6 PUFA, linoleic acid (LA), constitutes the majority of PUFA intake in the western diet. LA is the precursor of the *n*-6 PUFA arachidonic acid (AA). A high LA intake is considered proinflammatory, however the evidence to support this is contradictory and not conclusive [27,34,35,36]. AA intake in the diet is low relative to LA intake, its metabolic precursor. However, AA is the most prevalent *n*-6 PUFA in inflammatory cell membranes and is the substrate for the synthesis of the proinflammatory eicosanoids, including prostaglandin E2 (PGE_2_) and 4-series leukotrienes, associated with inflammatory processes [27]. Despite this, studies investigating the impact of AA on inflammatory markers are inconclusive, and few human intervention studies have reported on the effect of dietary intake of AA on low-grade inflammation [37,38,39].

The *n*-3 PUFA, alpha-linolenic acid (ALA) is an 18-carbon *n*-3 essential fatty acid common in canola, soybean oil and some nuts, but in greatest concentrations in flaxseed and flaxseed oil [40]. ALA is elongated and desaturated to eicosapentanoic acid (EPA) and further to decosahexanoic acid (DHA), however the efficiency of this conversion has been debated [41]. Association studies between dietary intake of ALA and inflammatory markers suggest a modest anti-inflammatory effect of ALA [27,34,36,42,43,44].

The long chain *n*-3 PUFAs, EPA and DHA are found in seafood, especially oily fish and in some algal oils. Is it proposed that *n*-3 PUFAs affect inflammation mainly through altered eicosanoid production, but potentially also impacting cell signaling and gene expression [27,42]. When EPA and DHA are incorporated into human inflammatory cells, this is partly at the expense of AA, providing less substrate for eicosanoid production [27]. Culture systems, animal models and human intervention studies have been generally consistent in recognizing the anti-inflammatory actions of *n*-3 PUFAs [24,25,42,45,46,47,48].

## 4. Tumor Necrosis Factor-α

TNFα was the first cytokine associated with inflammation [20]. TNFα is overexpressed in the AT of obese individuals, with greater expression in visceral fat than in subcutaneous fat [1,49]. TNFα acts in a paracrine manner, suggesting that circulating TNFα levels may not be indicative of actual TNFα levels [50]. Despite this caution, elevated circulating levels of TNFα have been observed in the obese phenotype, which decrease with weight loss [51]. It was originally proposed that adipocytes were the principle source of raised TNFα levels in obesity, however, it is now well recognized that TNFα is abundantly produced by macrophages in the SVF [1,10,49,50]. TNFα has numerous effects in AT including regulating adipogenesis, lipid metabolism and insulin signaling [6,11]. In addition to its ability to increase other pro-inflammatory cytokines [49,52], TNFα has also been associated with a reduction in anti-inflammatory adipokines such as adiponectin [53,54]. Conclusively, TNFα plays a powerful role in regulating inflammatory pathways, favoring an overall inflammatory state [6].

The influence of TNFα on lipid metabolism is complex with the underlying mechanisms still unclear. However, Chen *et al.* in his review describes four signal pathways involved in TNFα-mediated lipid metabolism [55]. The effects of TNFα’s on lipid metabolism occur in different cells, tissues, and organs and include a number of metabolic processes. TNFα induces lipolysis, increasing free fatty acid (FFA) production. In addition, TNFα regulates cholesterol metabolism and other adipocyte-derived adipokines such as leptin, adiponectin, *etc.* which may also alter lipid metabolism [55].

A number of studies, in cell culture, rodent and human clinical models have confirmed the relationship between TNFα and lipid metabolism [55]. In clinical patients with dyslipidemia, TNFα levels were altered. Compared with healthy subjects, patients with hyperlipoproteinemia showed higher TNFα levels and raised total cholesterol (T-C), triglycerides (TAG), and LDL-C concentrations. Furthermore, after treatment with fenofibrate, T-C, TAG and VLDL-C decreased, which correlated with decreasing TNFα concentrations [56]. It has also been shown that cholesterol-lowering drugs such as simvastatin and atorvastatin decrease TNFα concentrations [57,58], and that blockading TNFα production improves lipid metabolism [59]. Lastly, administration of TNFα interferes with plasma lipid levels [55,60,61]. Feingold *et al.* have shown that an increase in hepatic VLDL-TAG secretion induced by TNFα is due to both the stimulation of hepatic *de novo* fatty acid synthesis and an increase in lipolysis [60].

### 4.1. TNFα and Dietary Fatty Acids

Several studies have investigated the effect of dietary fatty acids on TNFα concentrations and *TNFA* gene expression in cell, animal and human models (Table 1). Plasma TNFα levels and *TNFA* gene expression increased in 3T3-L1 adipocytes incubated with the SFA palmitic acid, whereas incubation with the MUFA, oleic acid and *n*-3 PUFA, DHA had no effect [62]. In rodent studies, supplementing the diet with *n*-3 PUFA decreased *TNFA* gene expression in mice [63]. In another study, rats were fed a standard diet (18% energy from protein, 76% as carbohydrate (CHO) and 6% of energy as lipid) or a high-fat cafeteria diet (9% energy as protein, 29% as CHO and 62% as lipid). The high fat diet increased both body weight and fat mass, however when these rats were supplemented with EPA they gained less weight, decreased their food intake and increased leptin production. *TNFA* gene expression was also increased by the high fat diet, but not in the rats supplemented with EPA [64].

Similar results were reported in human studies; Caucasians supplemented with ALA in the form of flaxseed oil in domestic food preparation for four weeks, experienced a 30% reduction in TNFα production. When these subjects were exposed to further supplementation with fish oil (9 g/day) for an additional four weeks, TNFα production was reduced by up to 70%. There was a significant inverse exponential relation between TNFα synthesis and mononuclear cell content of EPA [65]. Similarly, Endres *et al.* [66] used a radioimmunoassay to measure TNFα produced *in vitro* by stimulated peripheral-blood mononuclear cells. They reported that supplementation with *n*-3 PUFA (18 g/day) for six weeks decreased TNFα levels, but these levels returned to baseline levels once supplementation was stopped [66]. However, Grimble *et al.* demonstrated that the change in TNFα production in response to *n*-3 PUFA supplementation depended on the subjects’ plasma TNFα levels prior to supplementation, with subjects with lower pre-supplementation TNFα levels showing the greatest decrease in TNFα levels post-supplementation [67]. It is possible that the inherent inflammatory status, potentially due to a pre-existing condition such as obesity, could determine the extent of the inflammatory response to different dietary fatty acids. In addition to the independent influence of dietary fatty acids on TNFα production, variation in the *TNFA* gene may also contribute to the individual variability observed in TNFα production and *TNFA* gene expression.

### 4.2. *TNFA* Gene Variants, Obesity and Serum Lipids

Several SNPs have been identified in the promoter region of the *TNFA* gene, however the *TNFA* –308 G > A (rs1800629) and –238 G > A (rs361525) SNPs are most commonly associated with measures of adiposity, obesity risk and serum lipids (Table 2). The A allele of the functional –308 G > A SNP results in a 2-fold increase in *TNFA* transcription, with a subsequent increase in TNFα production [20]. Several studies have reported that carriers of the pro-inflammatory –308 A allele (AA and GA genotypes) reported a higher body mass index (BMI) and/or percent body fat than those with the GG genotype [68,69,70,71,72,73]. In a recent meta-analysis by Yu *et al.*, including 48 eligible studies, the –308 GA + AA genotypes were associated with an increased risk of obesity (OR, 1.19; 95% CI, 1.02–1.39) [21]. This result was consistent with the study by Sookoian *et al.*, who performed a meta-analysis of 31 observational studies with a total of 3562 individuals and showed that individuals with the GA + AA genotypes had a 23% elevated risk of obesity compared with the GG genotype (OR, 1.23; 95% CI, 1.05–1.45) [74]. In comparison to the –308 G > A SNP, only a few studies have investigated the association between the –238 G > A SNP and obesity (Table 2). In our laboratory it was found that black South African women with the –238 A allele had greater body fat % than those with the GG genotype [5]. To our knowledge only two papers have found an independent association between the –308 G > A SNP and serum lipid concentrations. In Caucasian men, the –308 A allele was associated with increased TAG [75], and in Polish Caucasian men and women the AA genotype was associated with lower high-density lipoprotein cholesterol (HDL-C) concentrations compared to the GG genotype [76].

**Table 1:** Dietary fatty acids and TNFα and IL-6 production.

	Study type	Dietary fatty acid	Effect on gene expression	Effect on plasma levels	Reference
**TNFα**					
3T3-L1 adipocytes. Incubation at 24 and 48 h with 50 or 500 μM fatty acid	Cell culture	SFA (PA)	Increase	Increase	[62]
		MUFA (OA)	No effect	No effect	
Human macrophages treated with *n*-3 PUFA	Cell culture	EPA & DHA	Decrease	Decrease	[77]
Male Wistar rats, high fat diet, 1 g/kg per day EPA, 5 weeks	Rodent	*n*-3 PUFA (EPA)	Prevent over expression	Not examined	[64]
NZB/NZW F1 Lupus-prone female mice, 10% fat, fed *ad lib* for lifespan	Rodent	*n*-3 PUFA	Decrease	Not examined	[63]
Caucasians. Supplemented normal diet with 18 g fish oil daily for 6 weeks	Human intervention (9)	*n*-3 PUFA		Decrease	[66]
Caucasians. Supplemented normal diet with 6 g fish oil daily for 12 weeks	Human intervention (111)	*n*-3 PUFA		Decrease in subjects with lower levels of TNFα before supplementation	[67]
Caucasians. Supplemented normal diet with flaxseed oil, and flaxseed oil and butter spread for 8 weeks. At week 4, diets were supplemented with fish oil, (1.62 g EPA, 1.08 g DHA)/day	Human intervention (28)	*n*-3 PUFA (EPA & DHA)		Decrease	[65]
**IL-6**					
3T3-L1 adipocytes. Incubation at 24 h with 250 μM fatty acid	Cell culture	SFA (PA)	Increase	Increase	[31]
		SFA (DA)	No effect	No effect	
		*n*-3 PUFA (DHA)	No effect	No effect	
Human macrophages treated with *n*-3 PUFA	Cell culture	EPA & DHA	Decrease	Decrease	[77]
Male c57bl/10sCn mice fed *ad lib* either high fat control diet (soybean oil) or a high PA diet for 16 weeks	Rodent	SFA (PA)	Increase	Not examined	[78]
Male Sprague-Dawley rats fed ad lib one of 3 diets: SFA, MUFA, or PUFA for 4 weeks	Rodent	SFA (coconut oil)	Not examined	Increase IL-6 release from adipocytes	[79]
		MUFA (olive oil)	Not examined	Decrease IL-6 release from adipocytes	
		PUFA (sunflower oil)	Not examined	Moderate IL-6 release from adipocytes	
Abdominally overweight Caucasians. Fed either SFA-rich diet (19% SFA and 11% MUFA) or MUFA-rich diet (20% MUFA and 11% SFA) for 8 weeks	Human intervention (20)	SFA	Increase *IL-6* gene expression		[80]
		MUFA	Decrease *IL-6* gene expression		
African Americans, Caucasians, Chinese and Hispanics men and women. Relationship between dietary intake (food frequency questionnaire) and biomarkers of inflammation and endothelial activation	Human (5677)	*n*-3 PUFA		Decrease in IL-6 levels	[81]
Relationship between plasma fatty acids and inflammatory marker levels	Human (1123)	*n*-3 PUFA (DHA)		Low plasma levels of DHA associated with increased IL-6 levels	[82]
		*n*-6 PUFA (AA)		Low plasma levels of AA associated with increased IL-6 levels	

The number of subjects (*n*) is in parentheses. *IL*, interleukin; SFA, saturated fatty acid; *TNFA*, tumor necrosis factor alpha; saturated fatty acid; MUFA, monounsaturated fatty acid; PUFA, polyunsaturated fatty acid; (*n*-3) PUFA, omega-3 polyunsaturated fatty acid; (*n*-6) PUFA, ALA, α-linolenic acid; LA, linoleic acid; EPA, eicosapentaenoic acid; DHA, docosahexaenoic acid. AA, arachidonic acid; DA, lauric acid.

**Table 2:** Studies investigating associations between *TNFA* and *IL-6* single nucleotide polymorphisms and obesity and serum lipids.

SNP	Study cohort	Genotype frequency	Result	Reference
***TNFA***				
**Obesity**				
*TNFA* –308 G > A	Caucasian N-W (154) and obese (154)	N-W, GG: 75.8%; GA + AA: 24.2% Obese, GG: 70.8%; GA + AA: 29.2%	G > A polymorphism by itself indicates only minor effect on obesity risk.	[71]
	Caucasian women (378)	GG: 72.2%; GA + AA: 27.7%	AA genotype more obese than GA and GG. Body fat of AA genotype increased by 1/3 compared with GA and GG genotype. In obese females BMI and body fat of AA genotype higher than GA and GG.	[70]
	Caucasian BMI < 27.3 (44), BMI 27.3–31.9 (44), BMI 31.9–36.5 (44) and BMI > 36.5 (44)	BMI < 27.3, GG: 75%; GA + AA: 25% BMI 27.3–31.9, GG: 68.2%; GA + AA: 31.8% BMI 31.9–36.5, GG: 77%; GA + AA: 33% BMI > 36.5, GG: 47.7%; GA + AA: 52.3%	Difference in allele frequencies of the SNP across BMI quartiles. Higher A allele frequency in highest BMI group. A allele carriers had higher BMI than G carriers.	[68]
	Caucasian (1392)	GG: 67.6%; GA + AA: 32.3%	Carriers of the A allele were more frequently obese than non-carriers (OR = 1.52).	[69]
	Caucasian normal wt. (79) and obese (115)	N-W, GG: 73.4%; GA + AA: 24.2% Obese, GG: 75.6%; GA + AA: 26%	No genotype difference between N-W and obese groups.	[83]
	Korean normal wt. (82) and obese (153)	N-W, GG: 82.9%; GA + AA: 17% Obese, GG: 84.3%; GA + AA: 15.7%	No difference in genotype between N-W and obese subjects. WHR was significantly lower in those with GA and AA genotype in obese women.	[84]
	Caucasian normotensive (113) and hypertensive (62)	Normotensive, GG: 84.8%; GA + AA: 15% Hypertensive, GG: 67.7%; GA + AA: 32.3%	No BMI difference for genotypes.	[74]
	Caucasian normal weight (64) and overweight (65)	Not shown	A allele associated with efficient lipid storage in overweight subjects.	[72]
	Caucasian men (262)	GG: 56.4%; GA + AA: 43.5%	A allele tendency to higher BMI value, WHR and abdominal diameter.	[73]
	Meta-analysis (48 eligible studies)		A allele associated with an increased risk of obesity (OR, 1.19; 95% CI, 1.02–1.39).	[21]
*TNFA* –238 G > A	N-W (107) and obese (120) black, and N-W (89) and obese (62) white SA women	Black, GG: 60%; GA: 40%; AA: 0%; A allele: 20% White, GG: 78.5%; GA: 21%; AA: 0.5%; A allele: 11%	Black women with the A allele had a greater body fat % than those with the GG genotype.	[5]
*TNFA* –308 G > A & –238 G > A	Iranian men and women (BMI < 25, 25 ≤ BMI < 30, BMI ≥ 30) (239)	Under 18 years, BMI < 85%, GG: 80.8%; GA: 19.2% BMI > 85%, GG: 81.2%; GA: 12.5%; AA: 6.2%. Above 18 years, BMI < 25, GG: 89.3%; GA: 10.7% 25 ≤ BMI < 30, GG: 87.7%; GA: 12.3% BMI ≥ 30, GG: 80.4%; 15.2%; 4.3%	A allele had no association with BMI.	[85]
	Caucasian and African-American non-diabetics (424)	–308 G > A BMI < 25, GG: 73.2%; GA: 22.5%, AA: 4.3% BMI 25–29.9, GG: 66.7%; GA: 27%, AA: 6.3% BMI 30–40, GG: 67.2%; GA: 30.2%; AA: 2.6% BMI > 40, GG: 73.4%; GA: 24.3%; AA: 2.4% –238 G > A BMI < 25, GG: 90.4%; GA: 9.1%, AA: 0.5% BMI 25–29.9, GG: 90.5%; GA: 7.9%, AA: 1.6% BMI 30–40, GG: 85.9%; GA: 14.1% BMI > 40, GG: 91.1%; GA: 8.9%	A allele had no association with BMI.	[86]
**Serum lipids**				
*TNFA* –308 G > A	Caucasian obese women (136) and obese men (34)	Obese women, GG: 57.3%; GA + AA: 42.6% Obese men, GG: 64.7%; GA + AA: 35.3%	AA genotype had lower HDL cholesterol than the GG genotype.	[75]
	Caucasian obese men (38) and obese women (83)	Obese men, GG: 50%; GA + AA: 50% Obese women, GG: 54.2%; GA + AA: 45.7%	A allele carriers in men had significantly increased levels of TG, FFAs and fasting glucose.	[76]
***IL-6***				
**Obesity**				
*IL-6* –174 G > C	Finnish men and women (1334)	GG: 19.3%; GC: 51.3%; CC: 29.3%	In men BMI was higher in the –174 CC genotype compared to GC and GG	[87]
	Meta-analysis (48 eligible studies)		The C allele was associated obesity when using allelic comparisons, the recessive genetic model and the dominant genetic model with OR (95% CI) of 1.95 (1.37–2.77), 1.44 (1.15–1.80), and 1.36 (1.16–1.59), respectively.	[21]
	Meta-analysis Caucasians, diabetic and non-diabetic (25635)		No evidence for association between –174 G > C polymorphism and BMI.	[88]
*IL-6* –174 G > C & IVS3 +281 G > T and IVS4 +869 A > G	Health men (980) and women (2255) and Meta-analysis (26944)		No association between –174 G > C polymorphism and adiposity. Tallele of the IVS3 +281 G > T polymorphism associated with adiposity when part of a haplotype.	[89]
*IL-6* –174 G > C IVS3 +281 G > T & IVS4 +869 A > G	N-W (108) and obese (124) black, and N-W (89) and obese (63) white SA women	-174 G > C N-W black: GG: 97%; GC: 3% Obese black: GG: 95%; GC: 3%; CC: 2% N-W white: GG: 30%; GC: 58%; CC: 11% Obese white: GG: 32%; GC: 46%; CC: 22% IVS3 +281 G > T N-W black: GG: 54%; GT: 38%; TT: 8% Obese black: GG: 55%; GT: 37%; TT:9% N-W white: GG: 35%; GT: 48%; TT: 17% Obese white: GG: 30%; GT: 51%; TT: 19% IVS4 +869 A > G N-W black: AA: 51%; AG: 39%; GG: 10% Obese black: AA: 54%; AG: 40%; GG: 6% N-W white: AA: 42%; AG: 57%; GG: 1% Obese white: AA: 37%; AG: 63%	The IVS4 +869 G allele was also associated with greater waist and fat mass in black women.	[90]
**Serum lipids**				
*IL-6* –174 G > C	Caucasian men (245) and women (252)	Women, GG: 28%; GC: 47%; CC: 24% Men, GG: 30%; GC: 46%; CC: 24%	The CC genotype was associated with lower levels of T-C and LDL-C in women.	[91]
	Finnish men and women (1334)	GG: 19.3%; GC: 51.3%; CC: 29.3%	In men, serum T-C and LDL-C was higher in –174 GG than in the GC or CC genotype.	[87]
	Spanish Caucasian men (15) and women (17)	GG: 25%; GC: 40.6%; CC: 34.4%	G allele associated with high carriers TAG, VLDL-C and slightly lower HDL-C compared to the C allele.	[92]
	Finnish men and women (2228)	GG: 20.8%; GC: 50.4%; CC: 28.8%	In men for HDL cholesterol was higher for –174 GG compared to GC or CC.	[93]
*IL-6* -174 G > C, IVS3 +281 G > T & IVS4 +869 A > G	N-W (108) and obese (124) black, and N-W (89) and obese (63) white SA women	–174 G > C N-W black: GG: 97%; GC: 3% Obese black: GG: 95%; GC: 3%; CC: 2% N-W white: GG: 30%; GC: 58%; CC: 11% Obese white: GG: 32%; GC: 46%; CC: 22% IVS3 +281 G > T N-W black: GG: 54%; GT: 38%; TT: 8% Obese black: GG: 55%; GT: 37%; TT:9% N-W white: GG: 35%; GT: 48%; TT: 17% Obese white: GG: 30%; GT: 51%; TT: 19% IVS4 +869 A > G N-W black: AA: 51%; AG: 39%; GG: 10% Obese black: AA: 54%; AG: 40%; GG: 6% N-W white: AA: 42%; AG: 57%; GG: 1% Obese white: AA: 37%; AG: 63%	The IVS3 +281 T allele had lower TAG concentrations than the GG genotype in white women.	[90]

Genotype frequency is expressed as a percentage. The number of subjects (*n*) is in parentheses. *IL*, interleukin; SFA, *TNFA*, tumor necrosis factor alpha; N-W; Normal-weight; WHR, waist-hip ratio; TAG, triglycerides; T-C, total cholesterol; HDL-C, high-density lipoprotein cholesterol; LDL-C, low-density lipoprotein cholesterol; T-C:HDL-C ratio, total cholesterol: high-density lipoprotein cholesterol ratio.

Not all studies have however shown an association between the –308 G > A and –238 G > A SNPs and obesity [3,4,74,83,84,85,86]. Further, to our knowledge, no independent associations have been reported between the –238 G > A SNP and serum lipid concentrations. While the A allele of the –308 G > A and –238 G > A SNPs appear to be associated with the obese phenotype and serum lipid concentrations, it is highly likely that genetic variation in the *TNFA* gene may provide only a partial explanation with regards the inter-individual variability observed and the heterogeneity of the results in these studies. Other variables such as ethnicity, gender, diet, lifestyle and environmental factors may modulate these associations and contribute to the different results observed.

### 4.3. *TNFA* Gene and Diet Interactions on Obesity and Serum Lipids

**Figure 2:**
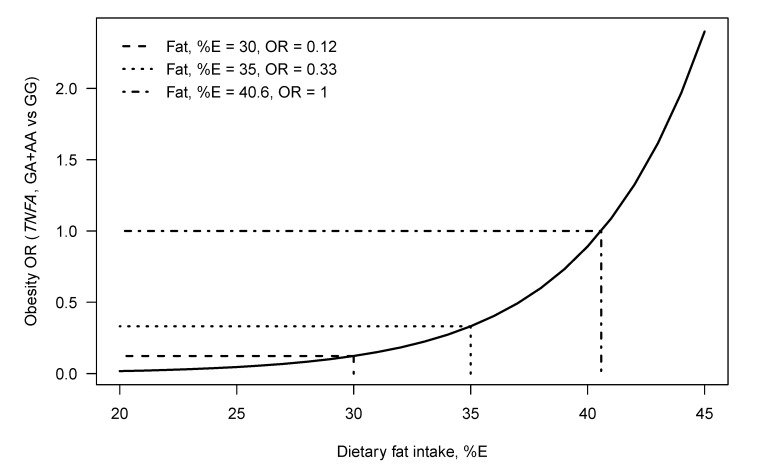
The modeled relationship between the odds of being obese (odds of being obese *vs*. being normal weight), *TNFA*–308 genotype and dietary fat intake (%E) for black SA women. The curve gives the modeled obesity OR for genotype GA+AA *versus* genotype GG, at each fat intake (%E). Lines show the total dietary fat intake (%E) of equal odds (OR = 1, for the genotype groups), namely 40.6 (%E), the OR for fat intake = 30 (%E) namely 0.12 and the OR for fat intake = 35 (%E), namely 0.33 [3].

The *TNFA* –308 G > A and –238 G > A SNPs have been shown to modulate the relationship between dietary fat intake on obesity and serum lipid profiles in different populations (Table 3). Despite the many studies showing independent association between the *TNFA* SNPs and obesity (Table 2), to our knowledge only two studies have investigated diet–gene interactions. Nieters *et al.* found that German Caucasian men and women with the –308 A allele, who were in the highest tertile for intake of the *n*-6 PUFAs LA and AA (%E), had an increased obesity risk [71]. More recently, we have reported that the odds of obesity for black South African (SA) women with the –308 A allele increased with total dietary fat intake (%E) [3] (Figure 2). This interaction was not observed in white SA women [4].

**Table 3:** Diet–gene interactions between *TNFA* and *IL-6* single nucleotide polymorphisms and dietary fat intake on obesity and serum lipids.

SNP	Study cohort	Genotype frequency	Diet assessment and fats	Diet-gene association	Reference
**TNFA**					
**Obesity**					
*TNFA* –308 G > A	Caucasian N-W (154) and obese (154)	Normal weight, GG: 75.8%; GA + AA: 24.2% Obese, GG: 70.8%; GA + AA: 29.2%	Food frequency questionnaire measured energy and dietary fatty acid intake.	A allele carriers had increased obesity risk with increasing intake of linoleic and arachidonic acid.	[71]
	Black South African N-W (105) and obese (118)	Black, GG: 69%; GA: 28%; AA: 3%; A allele: 17%	Food frequency questionnaire measured dietary fatty acid intake.	Increasing dietary fat intake (%E) was associated with an increase in obesity risk in GA + AA genotype compared with the GG genotype.	[3]
*TNFA* –238 G > A	N-W (107) and obese (120) black, and N-W (89) and obese (62) white SA women	Black, GG: 60%; GA: 40%. A allele: 20% White, GG: 78.5%; GA: 21%; AA: 0.5%; A allele: 11%	Food frequency questionnaire measured dietary fatty acid intake.	In black women: With increasing total fat and SFA intake (%E), adiposity increased for the GA genotype, but not the GG genotype. With increasing MUFA (%E), weight increased for the GA genotype, but not the GG genotype.	[5]
**Serum lipids**					
*TNFA* –308 G > A	Black South African N-W (105) and obese (118)	Black, GG: 69%; GA: 28%; AA: 3%; A allele: 17%	Food frequency questionnaire measured dietary fatty acid intake.	With increasing ALA intake (%E) the T-C:HDL-C ratio increased for the GG genotype and decreased for the GA + AA genotype. With increasing PUFA intake (%E) LDL-C decreased for the GG genotype and increased for the GA + AA genotype with increasing relative PUFA (%E).	[3]
	Caucasian South African N-W (88) and obese (60) white SA women	White, GG: 56%; GA: 42%; AA: 2%; A allele: 23%	Food frequency questionnaire measured dietary fatty acid intake.	With increasing SFA intake (%E), serum T-C decreased for the GG genotype and increased for the GA + AA genotypes.	[4]
*TNFA* –238 G > A	N-W (107) and obese (120) black, and N-W (89) and obese (62) white SA women	Black, GG: 60%; GA: 40%. A allele: 20% White, GG: 78.5%; GA: 21%; AA: 0.5%; A allele: 11%	Food frequency questionnaire measured dietary fatty acid intake.	In black women, with increasing P:S ratio and *n*-6:*n*-3 PUFA ratio, HDL-C decreased, and T-C:HDL-C ratio increased in those with the GA genotype but not the GG genotype. In black women, with increasing n-3 PUFA intake T-C:HDL-C ratio decreased in those with the GA genotype, but not in those with the GG genotype. In white SA women, with increasing EPA (%E) intake, LDL-C decreased in the GG genotype but not the GA genotype.	[5]
*TNFA* –308 G > A & –238 G > A	Ethnoracially diverse Canadian diabetic men (53) and women (56)	–308 G > A GG: 63.3%; GA: 32%; AA: 0.05% –238 G > A GG: 75.2%; GA: 21.1%; AA: 0.04%	Three-day food record measured dietary fat intake.	PUFA intake was inversely associated with HDL-C in carriers of the -308 A allele, but not in those with the GG genotype. PUFA intake was positively associated with serum HDL-C in -238 Aallele carriers but negatively associated in the *GG* genotype.	[94]
*TNFA* –308 G > A & –238 G > A	Ethnoracially diverse Canadian healthy men (202) and women (393)	–308 G > A, 11% for A allele –238 G > A, 5% for A allele	Food frequency questionnaire measured dietary intake.	In individuals with the -308 GG & -238 GG genotypes, *n*-3, *n*-6 and total PUFA intake was positively associated with HDL-C in men and women, and an inverse relationship was observed among men carrying the -308 A & -238 GG genotypes.	[95]
***IL-6***					
**Obesity**					
*IL-6* –174 G > C	Obese Caucasians men (181) and women (541)	GG: 28.8%; GC: 50.2%, CC: 18%.	Test meal consisted of 95%E from fat, of which 60% SFA.	The ability to increase fat oxidation after a high fat load was increased in subjects with -174 C allele.	[96]
	737 Spanish men and women	GG: 37.6%; GC: 46.8%; CC: 15.6%	Three years diet intervention assigned to low-fat diet; Mediterranean diet supplemented with virgin olive oil or with nuts.	At baseline, the CC genotype was associated with higher measures of adiposity. After 3 years, CC subjects following the Mediterranean diet supplemented with virgin olive oil had the lowest weight gain.	[97]
*IL-6* –174 G > C, IVS3 +281 G > T & IVS4 +869 G > A	N-W (107) and obese (120) black, and N-W (89) and obese (62) white SA women	–174 G > C N-W black: GG: 97%; GC: 3% Obese black: GG: 95%; GC: 3%; CC: 2% N-W white: GG: 30%; GC: 58%; CC: 11% Obese white: GG: 32%; GC: 46%; CC: 22% IVS3 +281 G > T N-W black: GG: 54%; GT: 38%; TT: 8% Obese black: GG: 55%; GT: 37%; TT:9% N-W white: GG: 35%; GT: 48%; TT: 17% Obese white: GG: 30%; GT: 51%; TT: 19% IVS4 +869 A > G N-W black: AA: 51%; AG: 39%; GG: 10% Obese black: AA: 54%; AG: 40%; GG: 6% N-W white: AA: 42%; AG: 57%; GG: 1% Obese white: AA: 37%; AG: 63%	Food frequency questionnaire measured dietary fatty acid intake.	In white women, with increasing *n*-3 PUFA, EPA and DHA intake (%E), BMI decreased in those with the CC + GC genotypes. In white women with increasing *n*-6:*n*-3 PUFA ratio, BMI increased equally with each additional -174 C allele. In white women, with increasing ALA intake (%E), BMI decreased with each T allele; while with increasing *n*-6:*n*-3 PUFA ratio, BMI increased with each additional IVS3 +281 T allele. In black women, with increasing dietary fat intake, BMI decreased in those with the IVS3 +281 TT genotype and increased in those with the GG + GT genotype. In black women with increasing MUFA, BMI also decreased in those with the IVS3 +281 TT genotype, but the increase in those with the GG + GT genotype was not significant. In white women, with increasing ALA (%E) intake, fat mass increased in those with the IVS4 +869 AA genotype and decreased in those with the AG + GG genotype. Also in white women, with increasing *n*-3 PUFA (%E) intake, fat mass decreased in those with IVS4 +869 AG + GG genotype, while with increasing *n*-6:*n*-3 PUFA ratio, fat mass increased in those with the AG + GG genotype; compared to those with the AA genotype. In black women, with increasing total fat, MUFA, and SFA, intake (%E), BMI, (as well as weight, waist and fat mass) increased in those with the IVS4 +869 AA + AG genotype and decreased in those with the GG genotype.	[98]
**Serum lipids**					
*IL-6* –174 G > C	Spanish Caucasian men and women (32)	GG: 25%; GC: 40.6%; CC: 34.4%	Measured fasting and post-glucose load plasma lipids.	G allele carriers showed higher TAG and VLDL-C, and higher fasting and post-glucose load free fatty acids levels than C allele carriers.	[92]
*IL-6* –174 G > C, IVS3 +281 G > T & IVS4 +869 G > A	N-W (107) and obese (120) black, and N-W (89) and obese (62) white SA women	–174 G > C N-W black: GG: 97%; GC: 3% Obese black: GG: 95%; GC: 3%; CC: 2% N-W white: GG: 30%; GC: 58%; CC: 11% Obese white: GG: 32%; GC: 46%; CC: 22% IVS3 +281 G > T N-W black: GG: 54%; GT: 38%; TT: 8% Obese black: GG: 55%; GT: 37%; TT:9% N-W white: GG: 35%; GT: 48%; TT: 17% Obese white: GG: 30%; GT: 51%; TT: 19% IVS4 +869 A > G N-W black: AA: 51%; AG: 39%; GG: 10% Obese black: AA: 54%; AG: 40%; GG: 6% N-W white: AA: 42%; AG: 57%; GG: 1% Obese white: AA: 37%; AG: 63%	Food frequency questionnaire measured dietary fatty acid intake.	In white women, with increasing MUFA, and EPA intakes, TAG decreased with each –174 C allele, and with increasing n-3 PUFA intake (%E), HDL-C increased with each –174 C allele. With increasing EPA and DHA intake (%E), the T-C:HDL-C ratio decreased in those with the –174 CC compared to CG + GG genotype. In white women, with increasing *n*-3 PUFA intake (%E), T-C:HDL-C ratio decreased only in those with the IVS3 +281 TT genotype compared to TG + GG genotype, and with increasing ALA intake (%E), HDL-C increased significantly with each additional IVS3 +281 T allele. In white women, we found that with increasing EPA and DHA intake (%E) HDL-C decreased with each IVS4 +869 G allele; in those the minor homozygote GG, the decrease in HDL-C was significant. In black women, with increasing total fat intake (%E) and *n*-6:*n*-3 PUFA ratio, TAG (*P* = 0.049) increased with each IVS4 +869 G allele, this was also seen for T-C:HDL-C ratio (*P* = 0.029). For both scenarios, with increasing dietary fat intake, the serum lipid decreases in the major homozygotes, AA, and increases in minor homozygotes, GG.	[98]

Genotype frequency is expressed as a percentage. The number of subjects (*n*) is in parentheses. *IL*, interleukin; SFA, *TNAF*, tumor necrosis factor alpha; N-W; Normal-weight; TAG, triglycerides; T-C, total cholesterol; HDL-C, high density lipoprotein cholesterol; LDL-C, low density lipoprotein cholesterol; T-C:HDL-C ratio, total cholesterol: high density lipoprotein cholesterol ratio, SFA, saturated fatty acid; MUFA, monounsaturated fatty acid; PUFA, polyunsaturated fatty acid; P:S ratio, polyunsaturated fatty acid : saturated fatty acid ratio; (*n*-3) PUFA, omega-3 polyunsaturated fatty acid; (*n*-6) PUFA, omega-6 polyunsaturated fatty acid; (*n*-6):(*n*-3) PUFA ratio, omega-6:omega-3 polyunsaturated fatty acid ratio; ALA, α-linolenic acid; LA, linoleic acid; EPA, eicosapentaenoic acid; DHA, docosahexaenoic acid.

Only one study has reported a diet–gene interaction between the –238 G > A SNP, dietary intake and obesity (Table 3). We found that in black SA women, with increasing total fat, SFA and MUFA intake (%E), weight increased in those with the –238 GA genotype, but not the GG genotype [5]. In contrast, a number of studies have shown interactions between the –308 G > A and –238 G > A SNPs and dietary fat intake on serum lipid profiles (Table 3). In ethnically diverse diabetic Canadians, Fontaine-Bisson *et al.* reported that PUFA intake was inversely associated with HDL-C concentrations in –308 A allele carriers, but positively associated with HDL-C concentrations in –238 A allele carriers [94]. In a combined analysis in healthy non-diabetic Canadians, they also reported that in individuals with the –308 GA + AA and –238 GG genotypes, an inverse relationship was observed between HDL-C concentrations and *n*-3, *n*-6 and total PUFA intake [95]. 

More recently, we reported interactions between dietary fat intake and the *TNFA* –308 G > A and –238 G > A SNPs on serum lipid profiles in black and white SA women. With increasing dietary fat intake, serum lipids increased in black women with the –308 GA + AA genotypes; however, with increasing *n*-3 PUFA and ALA intake, total cholesterol:HDL-cholesterol ratio (T-C:HDL-C ratio) decreased in black SA women [3,4]. For the –238 G > A SNP, in black SA women with increasing polyunsaturated:saturated fat ratio and *n*-6:*n*-3 ratio, HDL-C concentrations decreased, and T-C:HDL-C ratio increased in those with the –238 GA genotype but not the GG genotype. However, with increasing *n*-3 PUFA, T-C:HDL-C ratio decreased in those with the –238 GA genotype, but not in those with the GG genotype. In white SA women, with increasing EPA intake, LDL-C concentrations decreased in those with the –238 GG genotype but not the GA genotype [5]. Interactions between the –238 G > A SNP and dietary fat intake on serum lipids differed depending on the type of dietary fatty acid consumed, and were not the same in black and white SA women suggesting that ethnicity as well as diet–gene interactions contribute to the complexity and heterogeneity observed within serum lipid profiles.

## 5. Interleukin-6

During an acute incident, the cytokine IL-6 acts as an anti-inflammatory cytokine, but in a chronic inflammatory condition, IL-6 is pro-inflammatory, as well as being a key regulator of hepatic C-reactive protein (CRP) production [99,100]. IL-6 is secreted by a variety of cells. However approximately 30% of total IL-6 is produced by AT and macrophages that have infiltrated WAT produce approximately 50% of AT derived IL-6 [99,100,101]. It is therefore not surprising that higher circulating concentrations of IL-6 have been associated with obesity, especially visceral fat deposition [102,103,104], and decrease in response to weight loss [102,105].

IL-6 is associated with lipid metabolism and plays a role in the development of atherosclerosis through a number of different mechanisms causing metabolic and endothelial dysfunction. [13,101]. IL-6 impairs insulin action, elevating lipolysis, and increasing FFA release [106]. The increase in FFAs reduces nitric oxide (NO) bioavailability and impairs vasodilation [107]. Insulin resistance and subsequent hyperglycemia also increases lipoprotein oxidation and subsequent increased expression of adipocytokines [108]. In rats administrated IL-6, serum TAG, T-C levels and hepatic TAG secretion increased [109]. Similarly, in human studies, increased IL-6 levels have been associated with an increase in serum TAG and FFAs, and low HDL-C concentrations [110,111].

### 5.1. *IL-6* and Dietary Fatty Acids

Several studies have investigated the effect of different dietary fatty acids on IL-6 production and *IL-6* gene expression in cell, animal and human models, with all studies showing similar results (Table 1). An *in vitro* study reported that IL-6 plasma levels and *IL-6* gene expression increased when 3T3-L1 adipocytes were incubated with the SFA, palmitic acid, whereas no effect was observed for lauric acid (C12:0) and the *n*-3 PUFA, DHA [31]. Similarly, macrophages treated with EPA or DHA showed a decrease in LPS stimulated *IL-6* mRNA and IL-6 production. In agreement with these findings, rats fed a diet high in *n*-6 PUFA-rich sunflower oil showed moderate IL-6 release from adipocytes, which was less than when fed a SFA-rich diet, but greater than when fed a MUFA-rich diet [79]. In addition, in mice fed a low fat diet or one of two high-fat diets, consisting of either unsaturated soybean oil or saturated palmitic acid for 16 weeks, MCP-1 expression in AT tissue increased for both high-fat diets compared to the low-fat diet. However, the high saturated fat diet also showed a three-fold increase in *IL-6* expression in AT not observed in the soybean oil diet [78].

In human studies (Table 1), increased long-chain *n*-3 PUFA intake and fish consumption were associated with decreased plasma IL-6 concentrations and other inflammatory markers (matrix metalloproteinase-3, CRP, soluble intercellular adhesion molecule-1) in men from the Multi-Ethnic Study of Atherosclerosis cohort [81]. Furthermore, Ferrucci *et al.* reported that plasma levels of PUFAs, especially *n*-3 PUFAs, were independently associated with lower levels of pro-inflammatory markers and higher levels of anti-inflammatory markers [82]. A controlled feeding trial investigating the effects of SFA and MUFA-rich diets on serum lipid concentrations and whole-genome microarray gene expression profiles of AT, found that consuming a SFA-enriched diet for eight weeks resulted in increased expression of genes involved in inflammatory processes in AT including *IL-6*, and NFκB signaling, whereas the MUFA-enriched diet led to a more anti-inflammatory gene expression profile, accompanied by a decrease in serum LDL-C concentration [80].

### 5.2. *IL-6* Gene Variants, Obesity and Serum Lipids

There is growing scientific evidence reporting associations between DNA sequence variants within the *IL-6* gene and increased risk of obesity and dyslipidemia (Table 2) [88,91,99,101,112,113]. The most frequently studied *IL-6* SNP is –174 G > C (rs1800795). This is a functional SNP, with most studies showing the –174 C allele to be associated with raised IL-6 and CRP concentrations in mostly Caucasian populations [21,91,114,115]. This SNP is rare in individuals of African ancestry, is not informative, and has therefore not been studied in these populations. Association studies between the –174 G > C SNP, obesity and dyslipidemia have yielded conflicting results. A recent meta-analysis found the –174 G > C SNP was associated with obesity [21]. However, this finding was not reproduced in two other meta-analyses [88,89]. The relationship between the –174 G > C SNP appears to be complex in that while the –174 C allele appears to be the risk allele associated with obesity [21], it is the –174 G allele that is associated with raised serum lipids concentrations [87,92,93], despite the –174 C allele being shown to be associated with raised IL-6 and CRP levels [21,91,114,115]. The lack of consistent results in these association studies may be due to interactions between multiple SNPs on the *IL-6* gene that may modulate these relationships. This was illustrated by Qi *et al.*, who found no independent association between the –174 G > C SNP and obesity, but did identify an association between an *IL-6* haplotype containing the –174 G > C SNP and adiposity in healthy American men and women [89].

A number of studies have reported associations between the –174 G > C SNP and serum lipid profiles (Table 2). Though not consistent for all the studies [93], for most, in Caucasian men and women the –174 G allele was associated with higher T-C, LDL-C, VLDL-C and TAG concentrations compared to the C allele [88,92,93]. Riikola *et al.* suggested several possible reasons that may explain the inconclusiveness of study results [87]. These include differences in body mass and body composition, as well as metabolic and pharmacological interference in the different study cohorts. They also suggested that the effect of IL-6 on serum lipids may differ depending on the phase of development of atherosclerosis. Age may also impact these associations as subtle allelic effects observable in young populations may be masked by stronger life-long diet and lifestyle covariates in older populations [88].

Preliminary results from our laboratory found no independent associations between the –174 G > C SNP and obesity or serum lipid profiles in white SA women. However, we did find associations between the less researched SNPs *IL-6* IVS4 +869 A > G (rs2069845) and IVS3 +281 G > T (rs1554606) and obesity and serum lipid profiles in black and white SA women. The IVS4 +869 G allele was associated with greater waist and fat mass in black SA women, and the IVS3 +281 T allele was associated with lower TAG concentrations in white SA women compared to those with the GG genotype [90] (Table 2).

### 5.3. *IL-6* Gene and Diet Interactions on Obesity and Serum Lipids

As previously described, studies have shown that dietary fat intake modulates the relationship between *TNFA* gene variants and obesity and serum lipid profiles (Table 3). However, to our knowledge, only two published studies have reported on the relationship between any *IL-6* SNP and dietary intake, and both studies investigated *IL-6* –174 G > C. Corpeleijn *et al.* reported that the ability to increase fat oxidation after a high fat load was increased in obese European Caucasians with the –174 C allele [96]. In Spanish men and women with a high CVD risk, the –174 CC genotype was associated with higher levels of adiposity at baseline, however after three years of nutritional intervention, those with the –174 CC genotype following a Mediterranean-style diet, had the greatest reduction in body weight [97].

To our knowledge only a single study has reported an interaction between the –174 G > C SNP and serum lipids levels. In this study, those individuals with the G allele had higher post-glucose load TAG and VLDL-C concentrations, and higher post-glucose load FFA levels than C allele carriers [92].

**Figure 3:**
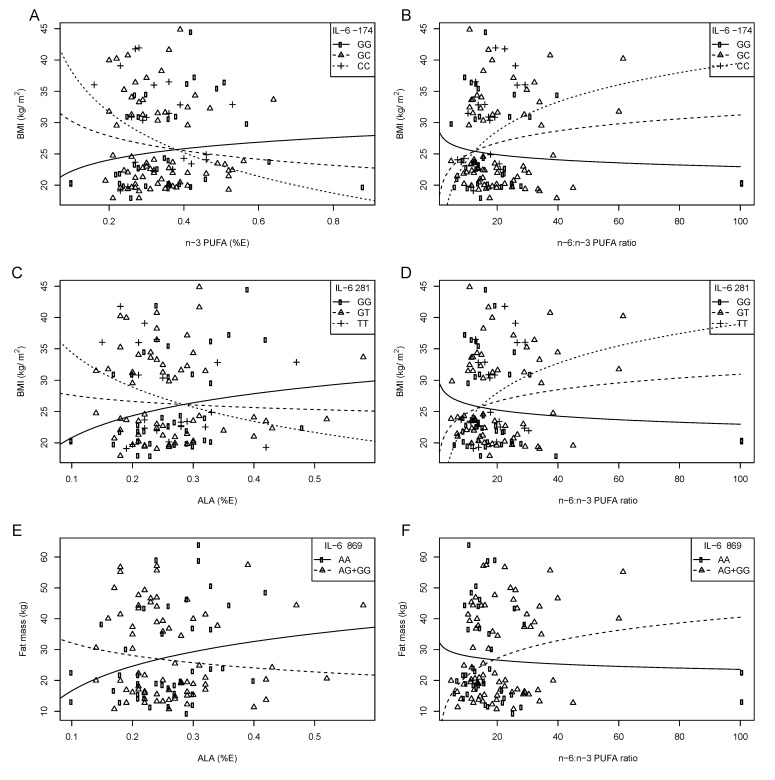
The relationship between BMI and fat mass, *IL-6* –174 G>C, IVS3+281 G>T and IVS4+869 A>G polymorphisms and dietary fat intake in white SA women. Symbols represent observed values for each woman. The lines are modeled relationships for a woman of average age (27.3 years) [98]. (**A**) With increasing *n*-3 PUFA intake (%E), BMI decreased in those with the –174 CC or GC genotypes. (**B**) With increasing *n*-6:*n*-3 PUFA ratio, BMI increased equally with each additional –174 C allele. (**C**) With increasing ALA intake (%E), BMI decreased with each additional IVS3+281 T allele. (**D**) With increasing *n*-6:*n*-3 PUFA ratio, BMI increased with each additional IVS3+281T allele. (**E**) With increasing ALA intake (%E), fat mass decreased in those with the IVS4+869 AG or GG genotype. (**F**) With increasing *n*-6:*n*-3 PUFA ratio, fat mass increased in those with the IVS4+869 AG or GG genotype; compared to those with the AA genotype.

Preliminary results from our laboratory have recently identified a number of interactions between three *IL-6* SNPs (*IL-6* –174 G > C, IVS3 +281 G > T, and IVS4 +869 A > G) and dietary fatty acids on obesity and serum lipid levels in both black and white SA women [98] (Table 3). In white SA women, with increasing *n*-3 PUFA intake and decreasing *n*-6:*n*-3 ratio, BMI decreased in those with the –174 C allele, IVS3 +281 T allele and IVS4 +869 AG genotype. In black SA women, with increasing dietary fat intake, adiposity decreased in those with the IVS3 +281 TT and IVS4 +869 GG genotypes and increased in the IVS3 +281 GT + GG and IVS4 +869 AA or AG genotypes (Figure 3). This figure illustrates that the type of fatty acid consumed in the diet interacts with *IL-6* genotypes to modulate measures of obesity.

When analyzing serum lipid profiles in white SA women, we observed that with increasing *n*-3 PUFA; TAG and T-C:HDL-C ratio decreased and HDL-C increased in those with the –174 C allele, and T-C:HDL-C ratio decreased in the IVS3 +281 TT genotype compared to the GG and GT genotype, with HDL-C increasing with each IVS3 +281 T allele. In contrast, HDL-C decreased with each IVS4 +869 G allele. In black SA women, with increasing total fat intake, TAG and T-C:HDL-C ratio increased in those with the IVS4 +869 G allele and decreased in the AA genotype [98]. These results suggest that both the quantity and quality of dietary fatty acids modulate the relationship between *IL-6* SNPs on measures of obesity and serum lipids, and that these effects may differ according to ethnic group.

## 6. The Role of Ethnicity and Gender as Confounders

It has been reported that while the prevalence of obesity may be high in a given population, the prevalence of obesity-associated comorbidities may also differ between ethnic groups [75,116,117,118,119,120]. As an example; while both African American and black SA women have a higher prevalence of obesity [117,121,122,123], they have less atherogenic lipid profiles than their white counterparts; characterized by low TAG, T-C, and LDL-C concentrations [118,124,125,126].

Differences between ethnicities comprise complex etiologies. Ethnicity incorporates a variety of different components including; genetic variation, diet and lifestyle, as well as cultural, behavioral and socio-demographic conditions [120]. In the example of ethnic differences in lipid profiles described above, ethnic variability may be observed in dietary intake, body fat distribution (VAT *vs.* SAT) [127,128], sequence variation in genes such as apolipoprotein E (*APOE*) [129], inflammatory gene expression (*TNFA* and *IL-6*) [130,131], and genotype frequencies. Genotype and allele frequencies of SNPs discussed in this review paper have been compared in Table 4 for European, British in England and Scotland, African, and African American populations. These SNPs are polymorphic in all these populations. However, there is a very low frequency of the *IL-6* –174 C allele in populations of African descent [132].

Of importance, dietary fatty acid intake may differ [133], as well as physical activity levels. In considering differences between ethnic groups resident in developed and developing countries, attitudes to food as well as the quality of the urban environment will also pay a role [120].

In a systematic review on the influence of ethnicity on the relationship between n-3 PUFA intake and CVD, Patel *et al.* concluded that ethnicity is a factor that accounts for inconsistencies in study results. Specifically, some of the effects of *n*-3 PUFA are limited to cultures with very high *n*-3 PUFA intake, and in turn, ethnicity moderates the efficiency with which PUFAs are derived from the diet [133]. Another key consideration in reviewing dietary intake and diet–gene interactions is the impact that certain genes have on dietary fatty acid metabolism, and how these may differ between ethnic groups. Of interest is the fatty acid desaturase gene (*FADS*), which codes for enzymes in PUFA metabolism. Lu *et al.* reported how genetic variation in the *FADS1* gene interacted with dietary intake of both *n*-3 and *n*-6 PUFA to affect T-C and HDL-C concentrations [134]. Furthermore, it has recently been shown that *FADS* SNPs altered the capacity of different ethnic groups to synthesize long-chain fatty acids [135,136]. Specifically, Sergeant *et al.* found that *FADS* genotype frequencies differed significantly between African Americans and European Americans, and SNPs in the *FADS* genes meant that African Americans were able to more efficiently convert the *n*-6 fatty acid LA to the proinflammatory fatty acid AA, resulting in higher circulating AA levels, and potentially a more deleterious impact of a diet high in LA in this ethnic group [136].

**Table 4:** *TNFA* and *IL-6* genotype and minor allele frequencies.

	Ensemble 1000 Genomes: phase 1			
	EUR	GBR	AFR	ASW
***TNFA* –308 G** **>** **A rs1800629**				
*GG*	0.75	0.80	0.81	0.87
*GA*	0.24	0.17	0.18	0.13
*AA*	0.02	0.03	0.00	0.00
A allele	0.14	0.12	0.10	0.10
***TNFA*–238 G** **>** **A rs361525**				
GG	0.87	0.81	0.93	0.90
GA	0.13	0.19	0.07	0.10
AA	0.00	0.00	0.00	0.00
A allele	0.07	0.10	0.03	0.05
***IL-6* –174 G** **>** **C rs1800795**				
GG	0.36	0.39	0.95	0.78
GC	0.44	0.42	0.05	0.21
CC	0.20	0.19	0.0	0.0
C allele	0.41	0.40	0.02	0.11
***IL-6* IVS3** **+281 G** **>** **T rs1554606**				
GG	0.34	0.36	0.48	0.39
GT	0.45	0.44	0.46	0.57
TT	0.20	0.19	0.05	0.03
T allele	0.43	0.41	0.28	0.32
***IL-6* IVS4** **+869 A** **>** **G** **rs2069845**				
AA	0.34	0.36	0.46	0.37
GA	0.45	0.44	0.47	0.57
GG	0.20	0.19	0.05	0.04
G allele	0.43	0.41	0.29	0.33

Population frequencies are from the Ensemble public database 1000 Genomes [137,138]. EUR, European; GBR, British in England and Scotland; AFR, African; ASW, Americans of African Ancestry in SW USA.

In this review we have identified for both the *TNFA* and *IL-6* genes, independent associations and diet–gene interactions that differed between ethnic groups. It is likely that the contributing factors described above may partly explain these differences. Like ethnicity, it is likely that the gender (sex) of study participants may impact genotype-phenotype interactions. A number of studies have observed gene–diet–gender interactions, whereby an interaction was identified in one sex, but not another. For example, the FINGEN study, which examined the effect of long chain *n*-3 PUFA supplementation and *APOE* genotype on plasma lipids, reported greater TAG lowering effects following dietary intervention in APOE4 males than in females [139]. Further, results from the Framingham Heart Study showed that dietary PUFA intake modulates the effect of the apolipoprotein A1 (*APOA1*) –75 G > A polymorphism (rs670) on plasma *HDL-C* concentrations in women but not in men [140]. In addition, Phillips *et al.* have shown that the *TCF7L2* rs7903146 polymorphism influences MetS risk, and is impacted by both gender and dietary SFA intake [141]. Phillips *et al.* have also identified associations between genetic variants of the apolipoprotein B and *APOA1* gene and MetS risk, however the modulation of MetS risk by dietary fat intake observed in the entire cohort was observed in the male high-fat consumers only [142].

## 7. Conclusions

This review suggests that DNA sequence variations in genes involved in inflammation may interact with environmental exposures, such as dietary intake, to modulate an individual’s susceptibility to developing obesity and its comorbidities. In summary, dietary fatty acids, in particular SFAs and the *n*-3 and *n*-6 PUFAs, impact the expression of the cytokine genes *TNFA* and *IL-6*, and alter TNFα and IL-6 production. In addition, sequence variants in these genes have also been shown to alter their gene expression and plasma levels, and are associated with obesity, measures of adiposity and serum lipid concentrations. When interactions between dietary fatty acids with *TNFA* and *IL-6* SNPs on obesity and serum lipid were analyzed, it became evident that both the quantity and quality of dietary fatty acids modulate the relationship between *TNFA* and *IL-6* SNPS on obesity and serum lipid profiles, thereby impacting the association between phenotype and genotype. The inter-individual variability in the obese phenotype and the inconsistencies in study results may be better understood by researching these diet–gene interactions more extensively.

Although this review focused on only two of the inflammatory cytokines, many other adipokines and chemokines have been associated with obesity (e.g., adiponectin, IL-1, IL-10, and MCP-1), and are sensitive to dietary fatty acid intake and should be studied further. In addition, it is also important to unravel the molecular mechanisms that govern the impact of dietary fatty acids on inflammation, which remain unclear and appear to act via multiple pathways. While it is known that different groups of dietary fatty acids (SFA, MUFA, and *n*-3, *n*-6 and *n*-9 PUFA) differ in their effect on inflammatory gene expression and plasma levels [27], these results are not conclusive, especially with regards the *n*-6 PUFA class of fatty acids which have at times been shown to act in both a pro- and anti-inflammatory manner, and appear to be modulated by gene variants.

The nutrigenetic interactions described in this review are complex and it is difficult to assess the magnitude of their impact in managing an individual’s diet. Furthermore, the inter-ethnic variability reported should caution us with regards singular dietary recommendations, as it cannot be assumed that fatty acids and other nutrient metabolism is uniform for all populations. In addition to diet–single polymorphism interactions, it is also important to understand the combined effect of a number of polymorphisms on the same or different genes interacting with the environment. For this reason, the identification and analysis of haplotype-nutrient interactions may provide additional insights in future research. The future study of nutrigenomics offers the opportunity to clarify the underlying molecular mechanisms governing the interactions between dietary fatty acids and the inflammatory phenotype, potentially elucidating the observed differences between different ethnic groups and genders in developing population-specific dietary recommendations.
